# Suicide attempts and related factors in patients admitted to a general hospital: a ten-year cross-sectional study (1997-2007)

**DOI:** 10.1186/1471-244X-11-51

**Published:** 2011-03-31

**Authors:** Jesús Alberdi-Sudupe, Salvador Pita-Fernández, Sonia M Gómez-Pardiñas, Fernando Iglesias-Gil-de-Bernabé, Jorge García-Fernández, Gonzalo Martínez-Sande, Sara Lantes-Louzao, Sonia Pértega-Díaz

**Affiliations:** 1 Department of Psychiatry, A Coruña Hospital, UPIE Planta Baja, Hospital de Oza, As Xubias, 84, 15006 A Coruña, Spain; 2 Clinical Epidemiology and Biostatistics Unit, A Coruña Hospital, As Xubias, 84, 15006 A Coruña, Spain

## Abstract

**Background:**

Suicide and suicide attempts represent a severe problem for public health services. The aim of this study is to determine the socio-demographic and psychopathological variables associated with suicide attempts in the population admitted to a General Hospital.

**Methods:**

An observational-descriptive study of patients admitted to the A Coruña University Hospital (Spain) during the period 1997-2007, assessed by the Consultation and Liaison Psychiatric Unit. We include n = 5,234 admissions from 4,509 patients. Among these admissions, n = 361 (6.9%) were subsequent to a suicide attempt. Admissions arising from a suicide attempt were compared with admissions occurring due to other reasons.

Multivariate generalised estimating equation logistic regression models were used to examine factors associated with suicide attempts.

**Results:**

Adjusting by age, gender, educational level, cohabitation status, being employed or unemployed, the psychiatric diagnosis at the time of the interview and the information on previous suicide attempts, we found that the variables associated with the risk of a suicide attempt were: age, psychiatric diagnosis and previous suicide attempts.

The risk of suicide attempts decreases with age (OR = 0.969). Psychiatric diagnosis was associated with a higher risk of suicide attempts, with the highest risk being found for Mood or Affective Disorders (OR = 7.49), followed by Personality Disorders (OR = 7.31), and Schizophrenia and Other Psychotic Disorders (OR = 5.03).

The strongest single predictive factor for suicide attempts was a prior history of attempts (OR = 23.63).

**Conclusions:**

Age, psychopathological diagnosis and previous suicide attempts are determinants of suicide attempts.

## Background

Suicide attempts constitute a serious problem for public healthcare services [1-3]. The data published on suicide attempts in Spain are scant [4,5], and no data are available on patients admitted to General Hospitals in our public healthcare setting after a suicide attempt.

Suicidal behaviour not only includes suicides, but also those suicide attempts which do not result in the patient's death. Mental phenomena, such as suicidal impulses or unconsummated ideations can also be included, as well as a wide range of behaviours which are pernicious to the patient's health without a previous explicit declaration of a suicidal intention [6,7].

A variety of factors are associated with an increased risk of suicide and attempted suicide [8], including psychiatric disorders [9-14], feelings of hopelessness and impulsivity [15,16], history of previous suicide attempts [9,17,18], age, sex and race [19-24], marital status [25,26], occupation [27], comorbidity [28,29], adverse childhood experiences [30], family history [31,32], and accessibility to weapons, especially firearms [33].

A recent paper [34] highlights the similarities and differences among heterogeneous groups of patients, some of whom committed suicide, and others who survived the attempt. The authors conclude that depressive symptoms are present in both groups as a shared variable.

Some of the individuals who attempt to commit suicide (by physically inflicting self-harm or ingesting a product at toxic dose levels) are taken to the Emergency Departments of General Hospitals, while others remain in their family or social environment after the attempt, with no specific health care; no official statistics on attempted suicides are available.

A proportion of these attending Accident and Emergency Departments will be admitted to the General Hospital's Medical and Surgical Departments, owing to the surgical lesions and medical comorbidity present after the suicide attempt. In this paper, we study patients admitted to a General Hospital, serving a health area with a population of 500,000, who required hospital admission, and for whom psychiatric assessment by the Consultation and Liaison Psychiatric Unit was requested, over an 11-year period (1997-2007). The aim of the present study was to determine the variables associated to suicide attempts in this group of admissions.

## Methods

### Setting and patients

The patients successively admitted to the A Coruña hospital between 1997 and 2007, with the exception of those admitted to the Department of Psychiatry, who required attention from the Consultation and Liaison Psychiatric Unit at the request of the appropriate Medical or Surgical Unit.

### Design

An observational-descriptive study of prevalence, using the hospital records of the patients attended. Admissions due to attempted suicide were compared with admissions for other reasons.

### Inclusion criteria

Patients admitted to the hospital during the study period, and for whom psychiatric assessment by the Consultation and Liaison Psychiatric Unit was requested.

### Exclusion criteria

Patients who were admitted directly to the Department of Psychiatry after assessment in the Emergency Department.

### Measures

For every patient included in the study, the following variables were assessed: age, gender, marital and cohabitation status, educational level, referring hospital department, profession, occupational situation, psychiatric diagnosis with the psychiatric interview (in line with DSM-IV and ICD-10 criteria), medical and surgical risk at the time of the psychiatric assessment, psychiatric personal and family background, therapeutic intervention during hospital admission, including the prescribed pharmacological treatment and psychotherapy.

### Sample size justification

The sample size (n = 5,234 admissions) makes it possible for the parameters of interest to be estimated with a confidence of 95% (α = 0.05) and a precision of ± 1.35%.

### Statistical analysis

The quantitative variables are expressed as mean ± standard deviation (SD); the qualitative variables are expressed as an absolute value (n) and the percentage, with the estimation of the 95% confidence interval (CI).

Comparisons for quantitative variables were made with the Student-T or Mann Whitney test, depending on which was appropriate subsequent to the verification of normality using the Kolgomorov-Smirnov test.

Qualitative variables associations were analysed using the Pearson Chi-Square test.

As some patients were admitted on multiple occasions, independent variables (*i.e. *demographic and clinical characteristics) may represent the same individual, giving rise to a lack of independence. In order to account for the correlated nature of these data, generalised estimating equations were used to assess associations between demographic and clinical characteristics and hospitalisation for suicide attempts [35]. Multivariate generalised estimating equation logistic regression models were used to examine factors associated with suicide attempts. The area under the ROC curve (AUC) was computed as a measure of model validity. All statistical analyses were performed using SPSS 17.0 and R 2.10.0, in addition to the Geepack package [36].

### Ethical-legal aspects

Authorisation was requested from the Clinical-Research Ethics Committee (CEIC) and confidentiality was preserved in accordance with the current Spanish Data Protection Law (15/1999).

## Results

### Descriptive study

The population under study consists of patients admitted to the Medical and Surgical Departments of the Complexo Hospitalario Universitario A Coruña (CHUAC), assessed by the Consultation and Liaison Psychiatric Unit of the Department of Psychiatry.

The analysis was conducted with hospitalisation as the unit of analysis. We include n = 5,234 admissions from 4,509 patients. This means that 725 (16.1%) were readmissions, some concerning the same patients.

Among the admissions occurring during the research period (1997 to 2007), n = 361 (6.9%) were subsequent to a suicide attempt. 58.4% of the suicide attempts were committed by women. Patients admitted subsequent to a suicide attempt were younger than those admitted for other reasons (42.5 ± 17.8 vs. 51.5 ± 18.1 years of age; p < 0.001)

The general features of the patients assessed, according to the presence or absence of suicide attempts, are shown in Table 1.

**Table 1:** General features of the episodes attended according to presence or absence of suicide attempt

	No suicide attempt		Suicide attempt		
	**Mean**	**SD**	**Mean**	**SD**	**p**

**Age**	51.5	18.1	42.5	17.8	< 0.001

	**n**	**%**	**n**	**%**	**p**

**Gender**					< 0.001
Men	2647	54.3	150	41.6	
Women	2226	45.7	211	58.4	
**Marital Status**					< 0.001
Single	1356	28.0	146	40.4	
Married	2529	52.3	138	38.2	
Separated	394	8.1	49	13.6	
Widowed	536	11.1	25	6.9	
Religious	7	0.1	2	0.6	
Others	18	0.4	1	0.3	
**Cohabitation status**					< 0.001
Alone	649	13.4	48	13.3	
Partner and children	2556	52.6	139	38.6	
Parents	673	13.9	81	22.5	
Alone with children	330	6.8	22	6.1	
With other relatives	472	9.7	55	15.3	
Institution	90	1.9	7	1.9	
Others	69	1.4	7	1.9	
Homeless	18	0.4	1	0.3	
**Employment**					< 0.001
No	3249	67.2	181	50.4	
Yes	1584	32.8	178	49.6	
**Educational level**					0.013
No Education	825	17.2	40	11.3	
Primary and Secondary Education	3587	74.8	281	79.2	
Higher and University Education	385	8.0	34	9.6	
**DSM-IV-TR grouped diagnosis with** **ICD-10 codes**					< 0.001
Schizophrenia and other Psychotic Disorders (333). F20-F29	274	5.7	44	12.3	
Mood or Affective Disorders (387). F30-39	533	11.1	106	29.5	
Personality Disorders (765). F60	259	5.4	87	24.2	
Other Diagnosis	3728	77.8	122	34.0	
**Previous suicide attempts**					< 0.001
No	4866	99.9	328	90.9	
Yes	7	0.1	33	9.1	

SD: standard deviation; DSM-IV-TR: Diagnosis and Statistical Manual of Mental Disorders, Fourth Edition, Text Revision; ICD: International Classification of Diseases

### Univariate analysis

In the univariate analysis, the variables significantly associated with suicide attempts were: age, gender, marital and cohabitation status, employment status, educational level, the psychiatric diagnosis according to DSM-IV-TR criteria (ICD-10 codes) and previous suicide attempts (Table 1).

Globally, the group of patients who attempted a suicide was significantly (p < 0.05) younger than the group of patients who had not (42.5 years of age vs. 51.5 years of age). There was also a higher percentage of women in the suicide attempt group than in the non-suicide attempt group (58.4% vs. 45.7%).

With regard to marital status, the percentages of single (40.4% vs. 28.0%) and separated individuals (13.6% vs. 8.1%) were also higher in the suicide attempt group than in the comparison group.

In relation to the cohabitation status variable, out of those patients who had made a suicide attempt, the percentage living with their parents (22.5% vs. 13.9%) or other relatives (15.3% vs. 9.7%) was higher than in the comparison group. The percentage of patients living with a partner and children was lower in the suicide-attempt group than in the non-suicide attempt group (38.6% vs. 52.6%).

The percentage of employment in the suicide attempt group is higher than in the other group of patients (49.6% vs. 32.8%), and higher educational levels were found in those attempting suicide attempt than in the other group.

With regard to grouped diagnosis, in accordance with the DSM-IV-R categories formulated with ICD-10 codes, a higher prevalence of psychiatric disorders was found in the suicide attempt group (12.3% vs. 5.7% for Psychotic Disorders, 29.5% vs. 11.1% for Mood of Affective Disorders, 24.2% vs. 5.4% for Personality Disorders).

There was also a higher prevalence of previous suicide attempts in the suicide attempter group than in the comparison group (9.1% vs. 0.1%).

In the univariate analysis, the variables not associated with the risk of attempted suicide were the province or domicile and the year of participation in the study.

### Multivariate analysis

A multivariate generalised estimating equation logistic regression model was performed, taking into account all the variables significantly associated with suicide attempts in the univariate analysis.

Adjusting by age, gender, educational level, cohabitation status, employment status, the psychiatric diagnosis at the moment of the interview, and the information on previous suicide attempts, we found that the variables associated with the risk of making a suicide attempt were: age, psychiatric diagnosis and previous suicide attempts. The area under the ROC curve (AUC), as a measure of model validity, was 0.805.

The risk of suicide attempt decreases with age (OR = 0.969), while psychiatric diagnosis was associated with a higher risk of suicide attempts: the highest risk was found for Mood or Affective Disorders (OR = 7.49), followed by Personality Disorders (OR = 7.31), and Schizophrenia and Other Psychotic Disorders (OR = 5.03).

The strongest single factor predictive of suicide attempt was a prior history of attempted suicide (OR = 23.63) (Table 2).

**Table 2:** Multivariate generalised estimating equation logistic regression model for predicting a suicide attempt adjusting for different variables and previous suicide attempts

	Regression coefficient	Standard error	p	OR	95% CI (OR)
**Intercept**	-2.316	0.287	< 0.001		
**Age**	-0.031	0.004	< 0.001	0.969	0.961-0.977
**Gender (Female)**	0.120	0.127	0.100	1.223	0.962-1.560
**Educational level**					
Illiterate				1	
Secondary education	0.154	0.204	0.448	1.167	0.783-1.739
Higher education	0.439	0.257	0.088	1.552	0.937-2.570
**Living alone**	0.051	0.180	0.778	1.052	0.739-1.497
**Employed**	0.145	0.188	0.441	1.156	1.800-1.670
**Psychiatric disorders**					
Psychotic disorders	1.615	0.202	< 0.001	5.030	3.387-7.470
Affective disorders	2.014	0.154	< 0.001	7.490	5.537-10.132
Personality disorders	1.989	0.169	< 0.001	7.310	5.249-10.180
Other diagnosis				1	
**Previous suicide attempt**	3.162	0.534	< 0.001	23.630	8.290-67.360

OR: Odds Ratio; CI: Confidence Interval

After stratifying by gender (Table 3), the variables associated with the presence of suicide attempt for both sexes were age, psychiatric diagnosis and previous suicide attempts.

**Table 3:** Multivariate generalised estimating equation logistic regression model for predicting a suicide attempt after stratifications according to gender

Men					
	**Regression coefficient**	**Standard error**	**p**	**OR**	**95% CI (OR)**

**Intercept**	-2.737	0.488	< 0.001		
**Age**	-0.032	0.006	< 0.001	0.968	0.956-0.980
**Educational level**					
Illiterate				1	
Secondary education	0.702	0.415	0.091	2.017	0.894-4.551
Higher education	0.959	0.470	0.041	2.609	1.039 -6.550
**Living alone**	0.266	0.235	0.258	1.304	0.823-2.068
**Employed**	-0.016	0.288	0.956	0.984	0.560-1.732
**Psychiatric disorders**					
Psychotic disorders	1.424	0.283	< 0.001	4.154	2.387-7.231
Affective disorders	1.822	0.259	< 0.001	6.182	3.722-10.269
Personality disorders	1.971	0.244	< 0.001	7.183	4.449-11.597
Other diagnosis				1	
**Previous suicide attempt**	3.977	0.659	< 0.001	53.387	14.662 -194.388

**Women**					

	**Regression coefficient**	**Standard error**	**p**	**OR**	**95% CI (OR)**

**Intercept**	-1.997	0.384	< 0.001		
**Age**	-0.031	0.006	< 0.001	0.969	0.958-0.980
**Educational level**					
Illiterate				1	
Secondary education	-0.065	0.248	0.790	0.937	0.576-1.525
Higher education	0.250	0.342	0.460	1.285	0.657-2.512
**Living alone**	-0.203	0.285	0.480	0.817	0.467-1.428
**Unemployed**	0.301	0.252	0.230	1.351	0.825 -2.213
**Psychiatric disorders**					
Psychotic disorders	1.854	0.286	< 0.001	6.385	3.645-11.184
Affective disorders	2.157	0.200	< 0.001	8.648	5.858-12.769
Personality disorders	2.026	0.240	< 0.001	7.582	4.738 -12.133
Other diagnosis				1	
**Previous suicide attempt**	2.830	0.656	< 0.001	16.947	4.684-61.313

OR: Odds Ratio; CI: Confidence Interval

**Figure 1:**
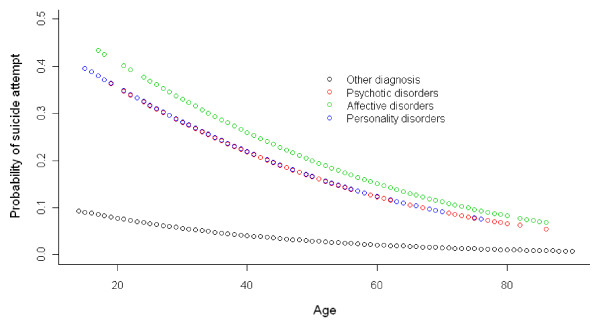
**Relationship between probability of suicide attempt, age and grouped diagnosis in women**.

**Figure 2:**
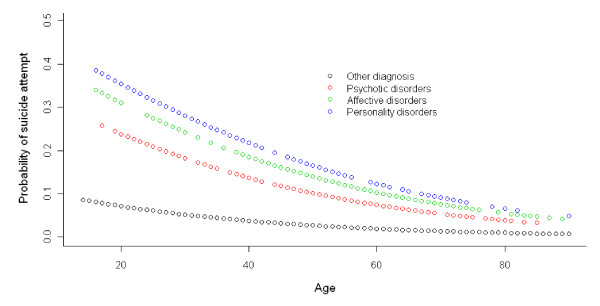
**Relationship between probability of suicide attempt, age and grouped diagnosis in men**.

The relationship between age, psychiatric diagnosis and the probability of suicide attempts according to gender in the multivariate analysis can be seen in Figures 1 and 2.

Among men, the psychiatric diagnosis with the highest risk for suicide attempts was Personality Disorders (OR = 7.18), followed by Affective Disorders (OR = 6.18) and Schizophrenia and Other Psychotic Disorders (OR = 4.15).

Among women, Affective Disorders (OR = 8.65), in the first place, followed by Personality Disorders (OR = 7.58) and Schizophrenia and Other Psychotic Disorders (OR = 6.38) were found to be associated to a higher risk of suicide attempts.

The strongest predictive factor of attempted suicide in both men and women was previous suicide attempts (Table 3).

## Discussion

The present study evidences that the variables associated to suicide attempts are age, psychiatric comorbidity and previous suicide attempts. These findings are referred to in the previous literature; hence, this study, carried out on hospital patients, reflects the consistency of the results in the previously described variables [2,37-39].

The association of psychiatric illness as a predictor of suicide attempts has been reported in the literature [9,10]. More than 90% of patients who attempt suicide have a major psychiatric disorder [11,12], and 95% of patients who committed a suicide attempt had a psychiatric diagnosis [13]. A very recent project [40] from the World Health Organisation (WHO) produced results on the association between the diagnosis of Mental Disorders and Suicidal Behaviours in several countries (including Spain), supported by worldwide data obtained from surveys in general populations (108,664 people from 21 countries on five continents answered the surveys). One of its conclusions is that the presence of a psychiatric diagnosis with DMS-IV Mental Disorder criteria is a strong predictor for the appearance of suicide ideations and behaviours, as well as for consummated suicide. This applies generally to both economically developed countries and to developing countries, except for one important difference: among the Mental Disorders associated with higher suicide risk, Affective Disorders stand out in developed countries, while the association is higher with diagnoses other than Affective Disorders, such as Impulse-Control Disorders, Substance Use Disorders and Post-Traumatic Stress Disorders, in developing countries.

In a large sample of patients in Sweden [10], the authors concluded that the category of psychiatric diagnosis coexisting with a suicide attempt is a variable which influences the future risk of consummated suicide. Planned reduction of the risk of future suicide among those patients requires implementing care plans, mainly in the first two years following the current suicide attempt, in particular among those patients diagnosed with Unipolar and Bipolar Affective Disorder, and Schizophrenia.

Although the risk of suicide increases with age, it has been demonstrated that in young adults, suicide attempts are more frequent than in older adults [19,20]. These findings, in relation with age, are consistent with those of the present study. In a study of 10,892 suicides and 57,439 attempted suicides among hospital-admitted individuals in 8 states, groups with high attempted suicide rates were teenagers, young adults, women, and people of Caucasian and Afro-American origin aged 25 to 44 years [19]. Another study in Korea with 344 attempted suicides found that a significantly higher number of women than men were admitted to emergency rooms due to attempted suicide during the study period [21]. In a random sample of 3021 adolescents aged 14-24 years, the females who attempted suicide showed suicidal thoughts and suicide attempts significantly more often, and suicide attempts at a far younger age than the males [23].

A systematic review of studies on the epidemiology of suicide published from 1997 to 2007 showed that non-fatal suicidal behaviours are more prevalent among women and young, unemployed, unmarried individuals, with low levels of education and suffering from a psychiatric disorder [1]. Our results are consistent in relation with age, gender and psychiatric disorders, but they do not coincide with the aforementioned study with regard to the variables of educational level and unemployment. In the univariate analysis we found a higher rate of suicide attempts among working populations, and with higher educational levels. The utilisation of health care services is probably related to socioeconomic status and educational level. Nonetheless, owing to the fact that we only have information on those patients who finally went to the hospital, we cannot make inferences regarding this point. In the multivariate generalised estimating equation logistic regression analysis, the association with educational level and employment disappears.

The variable most strongly associated with suicide attempts in the study is that of a prior history of attempted suicide. Ecological studies showed a positive correlation between rates of attempted suicide and suicide rates for both sexes among young people in Europe [41]. Prospective monitoring studies of patients hospitalised as a result of a suicide attempt show that the risk of suicide and all causes of mortality were very high immediately after discharge [9]. In a systematic review of data published on the following-up of patients subsequent to failed suicide attempts [42], the authors found that, in a one-year follow-up period subsequent to the failed suicide attempt, the repeat attempt rate was15%. There is a strong correlation between suicide attempt and subsequent death by suicide. In a study of a group of patients who attended the General Hospital after a self-harm episode, the presence of previous self-harm episodes is a risk factor for the onset of future suicidal behaviours [6]. This implies that outpatient care after attempted suicide is of paramount importance. Despite this fact, the identification of specific individuals who will go on to attempt and consummate suicide is currently unfeasible, owing to the scant sensitivity and specificity of the identification procedures available, and the low base rate of this behaviour [17,18].

Similar findings with respect to age, sex and previous suicide attempts have been reported in other publications. Thus, a study on a European level shows how the person-based suicide attempt rates were higher among women than among men and the highest person-based rates were found in the younger age groups [37]. Moreover, more than 50% of the individuals attempting suicide made more than one attempt [37]. Another study shows connection between suicide attempts and the individuals in the youngest age group, who reported suicidal ideation at baseline and psychiatric disorders, especially depression and drug abuse [38]. A systematic sample of 114 patients from consecutive cases of attempted suicide referred to a general hospital in Helsinki shows that a high proportion of individuals attempting suicide (82%) suffered from comorbid mental disorders [39].

In turn, in a population-based study conducted in Spain [2] among young women, mental disorders and psychiatric comorbidity and recent ideation of suicide were identified as high risk factors for an attempted suicide. Episodes of severe depression are the diagnosis representing the greatest risk in relation to the presence of the ideation of suicide and attempted suicide.

In relation with the differences found between men and women, the literature shows that a history of suicide attempt is significantly associated with any anxiety, personality, or substance use disorder among both men and women. However, in men, suicide attempts had a strong association with dependent personality disorder (adjusted odds ratio = 3.81; 95% CI = 1.14 to 12.73), whereas in women, suicide attempts had a strong association with antisocial personality disorder (adjusted odds ratio = 2.71; 95% CI = 1.72 to 4.25) [43].

## Limitations of the study

The present study has a number of shortcomings and biases, which we will now go on to point out. Attempted suicide is more difficult to study than consummated suicide, as there is a lack of generally approved reporting procedures for the former [44]. The possibility of a certain degree of under-reporting of attempted suicide in the present study cannot be excluded.

Another selection bias is associated with the use of hospital patients as a comparison group for identifying risk factors associated with suicide attempts.

The study is clearly conducted on admitted patients, some of whom are admitted owing to attempted suicide and others who are not (comparison group). Thus, the inference is not intended to be the general population, rather the sub-set of patients who are admitted to hospital. Nonetheless, given the magnitude of the problem in this sub-set of patients, we feel that it is relevant to be able to define the variables associated with the attempted suicide, while clearly accepting that these are patients who have been admitted for medical-surgical problems.

Given that the controls used in the hospital study are patients who also suffer from psychiatric disorders, we are aware of the presence of Berkson's bias [45,46], which occurs when hospital controls are used as a reference group. If the controls are hospitalised due to an exposure that is also related to the disease under study, then the measure of effect may be weakened; i.e. biased towards the null hypothesis of no association. This produces a systematically higher exposure rate among the comparison group, an therefore distorts the odds ratio with an underestimation of the suicide attempt risk. In this sense, we can confirm that the odds ratios associated with the mental disorder in the present study are lower than those that could be obtained in a population-base study. This has been confirmed by comparing the results of the present study with the population-based study by Gabilondo et al. (2007) [2], which shows how the presence of mental disorders is associated with a significant increase in attempted suicides, with higher odds ratio values than found in the present study, pointing towards an underestimation of the risk of attempted suicide depending on the type of controls used, as could have been expected.

Despite the underestimation of this effect, our findings are consistent with those from other studies and meta-analyses which show how psychiatric illness is a strong predictor of suicide [9,10,47]. A psychiatric disorder has also been identified as the strongest risk factor for attempted suicide [48-51]. More than 90 percent of patients who attempt suicide have a major psychiatric disorder [11,12]. Among patients with depression, a history of suicide attempts correlated most strongly with feelings of worthlessness [43]. Concurrent personality disorder was also strongly correlated with suicide attempts in depressed patients [43].

In relation to previous suicide attempts, we consider that one explanation for the extremely high association of previous suicide attempts with a current suicide attempt in this study could be that after the initial attempt, individuals may tend to stay with their family, being referred to the hospital more often after a subsequent suicide attempt. There may be an association between attending hospital after a suicide attempt and repeated suicide attempts. This effect, which is described as **s**urveillance bias or detection bias, occurs when one group is monitored more closely than the other group, and it may lead to an outcome being diagnosed more often in the more closely monitored group, but not because it actually occurred more frequently in that group. This association with previous suicide attempts was also identified in a nationwide study in Finland [9], and in a prospective study [17] in which the authors found that the strongest single factor for suicide is prior history of attempt suicide.

There may also be an information bias due to the fact that the diagnoses of mental disorders were not based on standardised diagnostic interview schedules, but instead were clinical diagnoses made by the psychiatrists treating the patients.

As a number of patients were admitted on multiple occasions, the results may be affected by the lack of independence among admissions. The analysis was conducted with hospitalisation as the unit of analysis, including 5,234 admissions from 4,509 patients. As previously mentioned, this means that 725 (16.1%) were readmissions, some concerning the same patients. To overcome this problem, the statistical analysis was performed using generalised estimating equations, in order to take into account the correlated nature of the data. More specifically, multivariate generalised estimating equation logistic regression models were used to identify those variables associated with suicide attempts [35]. This analysis takes into account the fact that the same patient could be admitted more than once during the study period. Generalised estimating equations are an extension of the generalised linear model, which takes into account this within-group correlation. They assume that the dependent variable of analyses can be expressed as a linear function of the independent variables through a monotonic differentiable link function, and that the variance of the dependent variable is a known function of its expectation. The parameters of the model are then estimated subsequent to the specification of the intra-cluster dependency correlation matrix.

In conclusion, our results show that the variables related to suicide attempt are age, psychiatric disorders and previous suicide attempts. This study in a hospital setting shows similar findings to population-based studies. Even though there is limited evidence as to the accuracy of screening tools for identifying suicide risk in the primary care setting, including tools for identifying those at high risk [52], physicians should remain alert to the possibility of suicide in high-risk patients, particularly if there is evidence of psychiatric disorder or if the patient has recently attempted suicide.

## Conclusions

The variables associated with suicide attempt are age, previous suicide attempts and certain grouped psychiatric diagnosis (Schizophrenia and Other Psychotic Disorders, Affective Disorders and Personality Disorders).

The risk of suicide attempts decreases with age. Psychiatric illness is a strong predictor of suicide attempt. The strongest single factor predictive of suicide attempts is a prior history of attempted suicide.

This study in a hospital setting shows similar findings to population based studies.

Even though there is limited evidence as to the accuracy of screening tools for identifying suicide risk in the primary care setting, including tools for identifying those at high risk, physicians should remain alert to the possibility of suicide in high-risk patients, particularly if there is evidence of psychiatric disorder or if the patient has recently attempted suicide

## Competing interests

The authors declare that they have no competing interests.

## Authors' contributions

JAS and SGP conceived the study and participated in its design. FIGB, JGF, GMS and SLL collaborated in the design of the study and data collection. SPF and SPD participated in the design of the study and performed the statistical analysis. JAS, SPF and SPD drafted the manuscript. All authors read and approved the final manuscript.

## Pre-publication history

The pre-publication history for this paper can be accessed here:

http://www.biomedcentral.com/1471-244X/11/51/prepub
